# Adaptive and maladaptive roles for ChREBP in the liver and pancreatic islets

**DOI:** 10.1016/j.jbc.2021.100623

**Published:** 2021-04-02

**Authors:** Liora S. Katz, Sharon Baumel-Alterzon, Donald K. Scott, Mark A. Herman

**Affiliations:** 1 Icahn School of Medicine at Mount Sinai, Obesity, Diabetes and Metabolism Institute, New York, New York, USA; 2 Division of Endocrinology and Metabolism, Duke University Medical Center, Durham, North Carolina, USA; 3 Duke Molecular Physiology Institute, Duke University Medical Center, Durham, North Carolina, USA

**Keywords:** ChREBP, carbohydrate metabolism, liver metabolism, pancreatic islet, fructose, transcription, metabolic disease, ChREBP, carbohydrate responsive-element binding protein, G6P, glucose-6-phospate, LID, low-glucose inhibitory domain, NAFLD, nonalcoholic fatty liver disease, SSB, sugar-sweetened beverage, T2D, type 2 diabetes, TKFC, triokinase and fmn cyclase

## Abstract

Excessive sugar consumption is a contributor to the worldwide epidemic of cardiometabolic disease. Understanding mechanisms by which sugar is sensed and regulates metabolic processes may provide new opportunities to prevent and treat these epidemics. Carbohydrate Responsive-Element Binding Protein (ChREBP) is a sugar-sensing transcription factor that mediates genomic responses to changes in carbohydrate abundance in key metabolic tissues. Carbohydrate metabolites activate the canonical form of ChREBP, ChREBP-alpha, which stimulates production of a potent, constitutively active ChREBP isoform called ChREBP-beta. Carbohydrate metabolites and other metabolic signals may also regulate ChREBP activity *via* posttranslational modifications including phosphorylation, acetylation, and O-GlcNAcylation that can affect ChREBP’s cellular localization, stability, binding to cofactors, and transcriptional activity. In this review, we discuss mechanisms regulating ChREBP activity and highlight phenotypes and controversies in ChREBP gain- and loss-of-function genetic rodent models focused on the liver and pancreatic islets.

Sugar consumption in the form of sucrose or high-fructose corn syrup has increased markedly in recent decades (1, 2). This is paralleled by increasing prevalence of obesity, type 2 diabetes (T2D), and cardiometabolic diseases including nonalcoholic fatty liver disease (NAFLD). However, the contribution of dietary sugar to cardiometabolic diseases remains controversial (3, 4, 5). Sugar-sweetened beverages (SSBs) are a major source of added dietary sugar (6), and SSB consumption consistently associates with indices of higher cardiometabolic risk suggesting that sugar consumption at rates commonly encountered in the population may be deleterious (3, 7, 8). However, other measures of dietary sugar exposure such as “total dietary sugar” often do not associate with cardiometabolic risk (3). Moreover, increased consumption of fruit, the most abundant source of natural sugar, is associated with improved health outcomes (9, 10, 11). Understanding molecular and physiological mechanisms that may contribute to the protective *versus* harmful effects associated with sugar consumption is of fundamental importance to explaining these epidemiological controversies and improving public health.

Carbohydrates including sugars are one of the three major classes of dietary macronutrients. Complex mechanisms and systems have evolved to sense carbohydrate consumption and regulate its metabolism and storage to meet our bodies’ long-term energetic and synthetic demands. Increases in circulating glucose following consumption of starch and sugar potently stimulate secretion of insulin from pancreatic beta-cells to coordinate systemic glucose homeostasis. Insulin increases glucose uptake in the muscle and adipose tissue for storage as glycogen and triglyceride, respectively. Insulin also suppresses glucose production by the liver and supports macromolecular anabolic programs such as protein synthesis throughout the body. Whereas glucose-mediated insulin secretion serves to integrate carbohydrate use systemically and maintain euglycemia, additional sugar-sensing systems exist within most cells to fine-tune cellular and tissue metabolic programs and responses independently of hormonal cues. In key metabolic cell types and tissues such as enterocytes, which are directly exposed to ingested nutrients, and the liver where ingested nutrients are delivered from the gut *via* the portal vein, these cellular nutrient-sensing mechanisms have further evolved to play key roles in the regulation of organ and systemic metabolism complementary to hormonal systems. Indeed, observations that high glucose levels in cell culture media regulated expression of glycolytic enzymes in isolated hepatocytes independently of insulin signaling led to the search for factors that mediated this cell autonomous metabolic regulation (12).

In 2001, Dr Kosaku Uyeda and colleagues discovered Carbohydrate Responsive-Element Binding Protein (ChREBP, also known as Mlxipl or MondoB), as the key factor that is activated by carbohydrate metabolites and transactivates genomic programs including glycolytic and lipogenic genes involved in the response to dietary and circulating sugars (12, 13). ChREBP has since been identified as a member of the Mondo family of basic helix-loop-helix transcription factors, which mediate an evolutionarily conserved and essential carbohydrate-sensing function across Animalia including *Drosophila* (14, 15). ChREBP is a specialized member of this family found in mammals and expressed at high levels in key metabolic tissues including the liver, white and brown adipose tissue, pancreatic islet cells, small intestine, and kidney with lower level expression in other tissues including skeletal muscle (13). MondoA (also known as Mlxip) is a glucose-sensing ChREBP homologue expressed ubiquitously in mammalian tissues and may fine-tune carbohydrate metabolism in these “non-metabolic” cell types (16, 17). Nevertheless, MondoA is expressed at significant levels in skeletal muscle and can regulate muscle and systemic glucose metabolism through its activity in this tissue (18). The ability of ChREBP and other Mondo family members to bind DNA and transactivate gene expression in response to carbohydrate metabolites requires dimerization with Max-like protein x (Mlx), another member of this transcription factor family (19).

Common genetic variants in the ChREBP locus including putative missense variants in ChREBP associate at genome-wide significance with numerous physiological traits and cardiometabolic risk factors including height, waist-to-hip ratio, circulating triglycerides, circulating HDL and LDL cholesterol, T2D, circulating gamma-glutamyl transferase, plasma c-reactive protein, and serum urate as well as many other diverse metabolic traits at near genome-wide significance (20, 21, 22, 23, 24, 25, 26, 27, 28, 29) (https://hugeamp.org/region.html?chr=7&end=73088873&phenotype=TG&start=7295752, accessed September 10, 2020). These associations indicate an important role for ChREBP in regulating human metabolic physiology and health. Investigators studying ChREBP have gained some appreciation of molecular and physiological mechanisms by which ChREBP links carbohydrate sensing to circulating lipids (detailed below). However, its role in regulating the balance of the aforementioned phenotypes and their significance to human health remain far less clear.

Our knowledge of the tissue-specific transcriptional targets and associated cellular and physiological mechanisms by which ChREBP mediates its pleiotropic metabolic effects remains poorly defined. In this review, we will discuss both adaptive and maladaptive functions of ChREBP in metabolic physiology guided by results in tissue-specific genetic gain- and loss-of-function models. We will limit our discussion to the role of ChREBP in the liver and pancreatic beta cells. Important roles for ChREBP have also been established in the intestine and white and brown adipose tissue. These aspects have been reviewed elsewhere recently (30, 31). We will focus on controversies and outstanding issues arising out of these genetic models that will be important to resolve through additional investigation. Resolution of these controversies will be essential to focus future work on identifying specific ChREBP targets and pathways that may be leveraged to prevent and treat cardiometabolic disease.

## Regulation of ChREBP activity by carbohydrate metabolites

ChREBP senses carbohydrate metabolites and regulates downstream cellular processes including metabolic pathways and cell proliferation *via* its effects on gene expression. As such, its activity is tightly regulated depending upon the status of carbohydrate fuel availability. The precise carbohydrate metabolites and the mechanisms by which these metabolites regulate ChREBP transcriptional activity remain controversial. An early model suggested that ChREBP transcriptional activity was primarily regulated by an effect of the pentose-phosphate metabolite, xylulose-5-phosphate, to activate a protein phosphatase and dephosphorylate ChREBP at cAMP-dependent protein kinase phosphorylation sites (12, 32). This dephosphorylation was proposed to cause ChREBP’s translocation into the nucleus and activation of its transcriptional targets. However, glucose responsiveness persists in mutant forms of ChREBP that cannot be phosphorylated by cAMP-dependent protein kinase (33). Moreover, mutant forms of ChREBP that are constitutively localized to the nucleus also retain carbohydrate responsiveness (34). Thus, nuclear localization, *per se*, is insufficient to activate ChREBP. As an alternative model, carbohydrate metabolites might directly bind and activate ChREBP through allosteric effects on the ChREBP protein itself. Glucose-6-phosphate (G6P) is the prime candidate for this activity (35, 36). The potential importance of G6P is supported by evidence that this metabolite also likely activates the ChREBP homologue, MondoA (17). Other metabolites are also implicated (37). The potential role of G6P as an allosteric regulator of ChREBP activity is supported by identification of five evolutionarily conserved “mondo conserved regions” (MCR) in the N-terminus of ChREBP and its Mondo homologues that comprise a “glucose-sensing module” composed of a “low-glucose inhibitory domain” (LID) and a “glucose-response activation conserved element” (14, 38). Sequence and modeling analysis suggests that elements in the conserved MCRs are similar to G6P-binding sites in other known G6P-binding proteins and may mediate allosteric activation (38). This model of “glucose sensing” is further supported by evidence that grafting the glucose-sensing module onto the Gal4/UAS reporter system is sufficient to recapitulate glucose sensing in heterologous cells, and this is independent of subcellular localization (14).

While allosteric activation of ChREBP might be required for its carbohydrate-sensing function, posttranslational modifications of ChREBP may alter protein stability, subcellular localization, protein–DNA or protein–protein interactions, all of which may alter the efficiency by which ChREBP transcribes its gene targets (32, 33, 35, 39, 40, 41, 42, 43, 44, 45, 46, 47, 48, 49, 50, 51, 52). For example, in the setting of hypoglycemia, ChREBP may be phosphorylated by cAMP-dependent protein kinase and AMP kinases, which decrease the ability of ChREBP to enter the nucleus and bind DNA (39, 40, 43). By contrast, when carbohydrates are abundant, ChREBP can be acetylated and O-GlcNAcylated, leading to increased binding affinities, stability, and transcriptional activity (49, 50, 51, 52). Several other posttranslational modifications with a variety of effects on ChREBP activity have been documented (Table 1). Another recently reported mode of regulation suggests that lipid droplets or lipid-droplet-associated proteins can sequester ChREBP and other members of the Mlx transcription factor family in the cytosol and limit its activation (53, 54). Many more mechanisms that reflect the metabolic state and function of particular cell types and modulate ChREBP activity are likely to be described in the future.

**Table 1:** Posttranslation modifications of ChREBP that regulate its activity

PTM	Location	Assigned function	Mediated by	Citation	Comment
PTMS that increase ChREBP activity					
Phosphorylation	Ser56	Enhanced transcriptional activity, enhanced nuclear retention	High glucose, kinase unknown	(135)	Several other phospho-acceptor sites identified^a^
Phosphorylation	Ser514	Enhanced O-GlcNAcylation of ChREBP	High glucose, kinase unknown	(50)	
Dephosphorylation	Ser196	Increased nuclear entry	Cytoplasmic PP2A, X-5P activated	(32, 41)	Near nuclear localization sequence
Dephosphorylation	Thr666	Increased DNA binding	Nuclear PP2A, X-5P activated	(32, 41)	In the basic helix-loop-helix DNA binding domain
Dephosphorylation	Ser566 (rat 568)	Increased DNA binding	Nuclear PP2A, X-5P activated	(32)	In the proline-rich region, near the DNA-binding domain
O-GlcNAcylation	Ser839, Thr517	Enhanced Mlx-heterodimerization, increased protein stability, enhanced transcriptional activity	OGT	(49, 50, 136)	
Acetylation	Lys672	Enhanced transcriptional activity	P300 HAT activity	(46, 47)	Glucose or EtOH diets
Proline hydroxylation	Pro536 Pro141	Enhanced transcriptional activity	proline hydroxylase	(48)	
PTMs that decrease ChREBP activity					
Phosphorylation	Ser196	Increased affinity to 14-3-3 and cytoplasmic retention	cAMP, PKA, PKG	(39, 40, 41)	
Phosphorylation	Thr666	Reduced transcriptional activity	cAMP, PKA	(41)	
Phosphorylation	Ser566 (rat Ser568)	Reduced transcriptional activity	Fatty acids, AMPK	(43)	
Phosphorylation	Ser140	Increased affinity to 14-3-3 and cytoplasmic retention	cAMP, PKA, PKG	(39, 40)	
Ubiquitination	Multiple regions	Degradation of ChREBP	DDB1 *via* CRY1; SMURF2	(43, 44, 47)	High-fructose diet protects against ubiquitination; ChREBP ubiquitination can reduce aerobic glycolysis, increase oxygen consumption, and decrease cell proliferation in cancer tissues and cell lines

ChREBP plays an important role in sensing fuel levels and regulating processes influencing metabolism, proliferation, and other cellular processes. Its activity is tightly regulated by fuel abundance and metabolic state. This is achieved in part through posttranslational modification of ChREBP, which may regulate ChREBP protein stability, subcellular localization, heterodimer formation, and binding to cofactors. The amino acid location of posttranslational modifications in this table is provided based on mouse ChREBP-alpha protein sequence.

a Ser25, Ser23, Ser56, Ser140, Thr147, Thr311, Ser366, Ser 524, Ser566, Ser614, Ser626, Ser643 were all found to be phosphorylated by MS analysis. Yet, except for Ser 56, no single-site mutant showed activation of ChREBP under low-glucose conditions or a loss of activation in the presence of high glucose.

**Figure 1:**
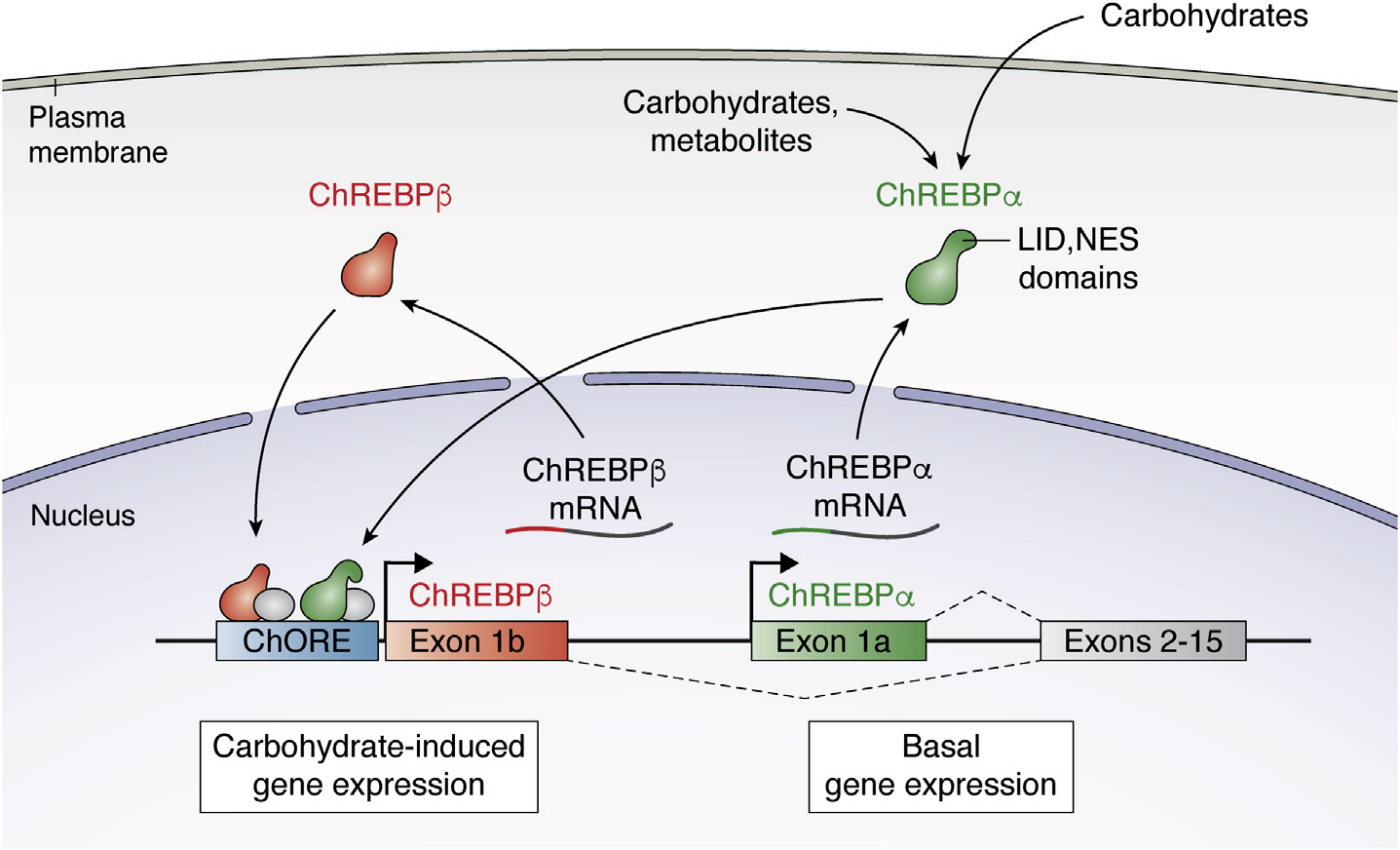
**Feed-forward regulation of ChREBP-beta expression.** Carbohydrate-derived metabolites activate ChREBP-alpha, which binds to carbohydrate response elements (ChoREs) in proximity to Exon 1b and induces transcription of ChREBP-beta, which is constitutively active as it is missing the LID domain. ChREBP-beta protein may activate its own expression by binding to the ChoREs near its promoter. A combination of metabolite-activated ChREBP-alpha and constitutively active ChREBP-beta mediates activation of other ChREBP target genes.

In addition to the canonical form of ChREBP now called ChREBP-alpha, a second form, called ChREBP-beta has been identified (55). The ChREBP-beta transcript is expressed from an alternative promoter and produces a protein that lacks the LID domain and nuclear export signals found in the canonical ChREBP-alpha isoform (55). Thus, ChREBP-beta activity is unrestrained by low glucose, constitutively nuclear, and orders of magnitude more transcriptionally potent than its larger counterpart. ChREBP-beta is induced by a powerful carbohydrate response elements located near the start site of the alternative ChREBP-beta promoter (55). Thus, increased carbohydrate metabolism activates the ChREBP-alpha isoform to initiate a vigorous feed-forward loop where the newly synthesized ChREBP-beta is capable of binding to the carbohydrate response element on its own promoter, producing more ChREBP-beta (56) (Fig. 1). Measurement of the expression the ChREBP-beta isoform in tissues is an excellent surrogate marker of ChREBP activity because of the marked and acute increase in ChREBP-beta expression upon ChREBP-alpha activation. ChREBP-beta expression is increased in the livers of humans with obesity and diabetes indicating that hepatic ChREBP activity is increased in association with metabolic disease (57, 58, 59). Some combination of ChREBP-alpha and ChREBP-beta mediates the effects of ChREBP activation on other ChREBP genomic targets. Recent work suggests that these isoforms may have distinct functions, and some of this evidence will be addressed in this review.

## Activation of hepatic ChREBP *in vivo*

ChREBP was discovered based on the ability of high-glucose cell culture media to regulate expression of the terminal enzyme in glycolysis, pyruvate kinase (Pklr), in isolated hepatocytes independently of insulin signaling (12). As a result, early studies on the physiological role of ChREBP focused on its function in the liver. Gain- and loss-of-function studies in both isolated hepatocytes and genetic mouse models confirmed an essential role for ChREBP in transactivating coordinate expression of glycolytic and lipogenic enzymes in response to carbohydrates (13, 60). As obesity and metabolic disease are commonly associated with increased hepatic lipogenesis, investigators have examined the function of hepatic ChREBP in a range of diet- and genetically induced obesity models. Here, we will describe evidence indicating that overnutrition with diets high in sugar and genetic models of obesity activate hepatic ChREBP, which transactivates expression of gene programs mediating both adaptive and maladaptive changes in metabolism. The dominance of adaptive *versus* maladaptive effects may depend on the degree and duration of ChREBP activity as well as the specific nutritional and genetic context.

Whereas high-glucose concentrations robustly activate ChREBP in isolated hepatocytes, hepatic ChREBP activity *in vivo* appears more responsive to sugars other than glucose (61, 62). Specifically, hepatic ChREBP is acutely and potently activated by fructose ingestion (62). Remarkably, global ChREBP knockout mice tolerate high-starch diets, but are intolerant to diets containing fructose (13). Global ChREBP knockout mice become moribund and die within 1 to 2 weeks when challenged with diets high in fructose. Gavaging mice with fructose acutely activates expression of ChREBP-beta and canonical ChREBP targets, and this does not happen when mice are gavaged with isocaloric glucose (62). Further supporting an important role of ChREBP in fructose metabolism, ChREBP coordinately regulates expression of all three fructolytic enzymes—ketohexokinase (KHK), aldolase b (ALDOB), and triokinase and fmn cyclase (TKFC) (13, 61, 62). Deficiency in either ALDOB or TKFC can cause fructose-associated cellular and organismal toxicities (63, 64). Thus, ChREBP appears important for adaptive responses to the consumption of dietary sugars containing fructose.

The potency of fructose over glucose to acutely activate ChREBP likely depends on the distinct biochemical pathways and enzymes by which fructose and glucose are metabolized within the liver. The liver and selected other tissues express a high-activity isoform of ketohexokinase (KHKc), which catalyzes fructose phosphorylation, the first step in fructose metabolism (65). This high-activity enzyme permits efficient first-pass extraction of ingested fructose in the liver. Substrate derived from fructose enters the triose-phosphate carbon pool and can be converted to glucose-6-phospate (G6P) *via* the gluconeogenic pathway or catabolized to pyruvate *via* glycolysis. Presumably, G6P or other key intermediary metabolites generated from fructose activate ChREBP. In contrast with fructose, glucose phosphorylation is catalyzed by glucokinase. In the liver glucokinase is sequestered in the nucleus in an inhibited state by the glucokinase regulatory protein (GCKR) (66). As a result, only a small fraction of ingested glucose is extracted by the liver first pass. This may limit hepatic ChREBP activation when glucose is ingested in isolation (62). While glucose gavage alone is insufficient to acutely activate hepatic ChREBP, glucose gavage can activate hepatic ChREBP when glucokinase is activated pharmacologically (62). Altogether, these results are consistent with *in vitro* data indicating that a hexose-phosphate in the glycolytic/gluconeogenic carbon pool, likely G6P itself, allosterically activates ChREBP.

Whichever metabolite can activate ChREBP, it does so no matter its original source. Indeed, activation of ChREBP is not limited to dietary sugars. Any carbohydrate precursor that can be avidly metabolized in the liver and increase the hepatic triose- or hexose-phosphate pool appears capable of activating ChREBP. As an example, glycerol, which is generated *via* lipolysis in the adipose tissue, is also efficiently extracted by the liver and rapidly phosphorylated by glycerol kinase (67). Administration of glycerol, like fructose, acutely and robustly activates hepatic ChREBP (62). Indeed, increased glycerol turnover that occurs due to increased adipose tissue lipolysis in obesity and diabetes may be a mechanism by which these conditions can activate hepatic ChREBP independently of dietary carbohydrate consumption (57, 58, 68, 69). Consistent with this hypothesis, circulating levels of the ChREBP-induced hepatokine FGF21 correlate with glycerol turnover in obese subjects (70). While ChREBP activity is increased in the livers of people with obesity and diabetes, whether this increase is on balance protective or harmful remains unclear.

## Hexose-phosphate and glucose homeostasis in ChREBP loss-of-function models

**Figure 2:**
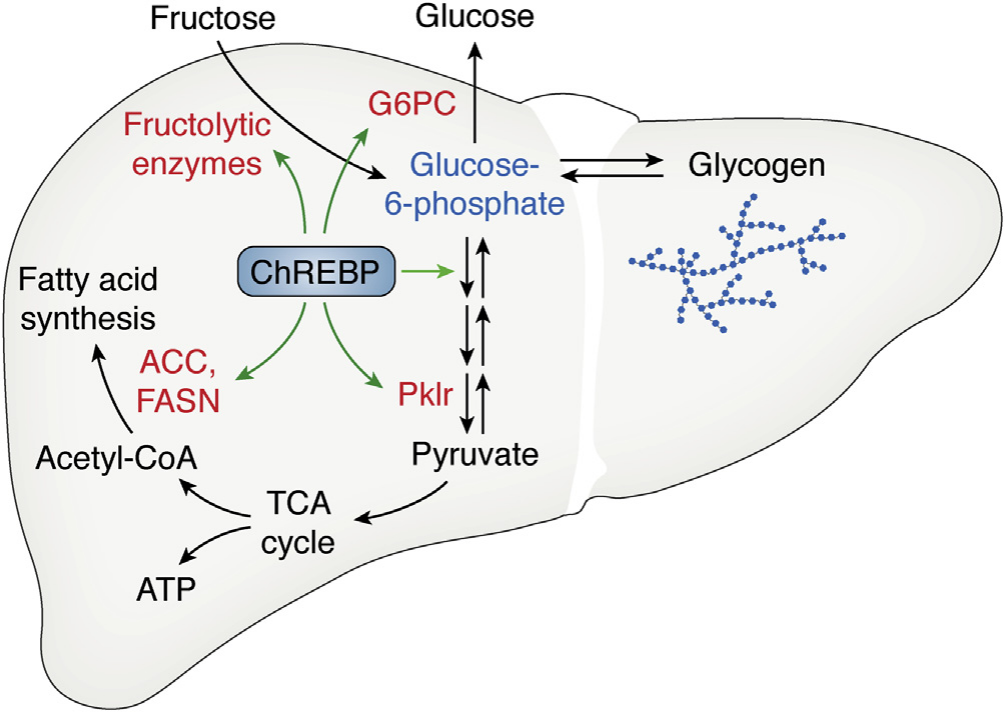
**ChREBP mediates G6P homeostasis.** Sugar consumption particularly with sugars containing fructose increases hepatocellular hexose-phosphate levels including G6P. G6P or a closely related metabolite activates ChREBP, which transactivates expression of enzymes involved in G6P disposal. This includes enzymes of glycolysis such as Pklr and enzymes of *de novo* lipogenesis including ACC and FASN. This also includes enzymes involved in glucose production such as G6PC. Catabolism of G6P either *via* glycolysis or glucose production reduces G6P inhibiting ChREBP and returning the system to a homeostatic equilibrium. Enzymes noted in *red* are ChREBP transcriptional targets.

Since ChREBP’s discovery, its role to coordinate carbohydrate metabolite availability with carbohydrate catabolism by enhancing glycolysis and lipogenesis has been well recognized. However, we and others also noted that ChREBP stimulates expression of genes involved in glucose production. This includes the terminal step in gluconeogenesis catalyzed by glucose-6-phosphatase (G6PC) (13, 62, 71, 72). The counterintuitive observation that ChREBP activates enzymes that utilize glucose and also enzymes that produce glucose leads to a mechanistic model that ChREBP’s essential cellular function is to mediate intracellular G6P homeostasis (Fig. 2) (62, 71). Thus, an increase in intracellular G6P activates ChREBP to mediate G6P disposal through glycolysis, lipogenesis, and, in capable tissues, glucose production to restore G6P levels and the hexose-phosphate pool to its homeostatic equilibrium.

Impeding this homeostatic mechanism by inactivating hepatic ChREBP leads to highly elevated hepatocellular G6P levels (13, 62). And since G6P potently activates glycogen synthase (73), this results in marked glycogen accumulation not unlike Glycogen Storage Disease Type 1a (GSD-1a), which is the result of loss-of-function mutations in G6PC (74). Indeed, hepatic ChREBP is highly active in the livers of mice with GSD-1a (74). Knockdown of hepatic ChREBP in a GSD-1a mouse model will also further limit G6P egress and exacerbate accumulation of glycogen (75). The marked accumulation of hepatic glycogen resulting from hepatic ChREBP knockout combined with fructose overfeeding causes structural hepatocellular injury associated with transaminitis that can be alleviated *via* pyruvate kinase overexpression (76). Thus, ChREBP activation mediates an adaptive response to sugar consumption to prevent glycogen overload and hepatic injury.

The molecular determinants of glucose production and “hepatic insulin resistance” are a topic of considerable controversy (77). Given robust induction of hepatic G6PC by fructose feeding, and reports that fructose feeding rapidly induces “hepatic insulin resistance” (78), our laboratory showed that fructose feeding, through activation of ChREBP and upregulation of G6PC, can drive glucose production (62). Moreover, ChREBP-mediated induction of G6PC remains intact in liver-specific Foxo1 knockout mice (62). Foxo1 is typically repressed by insulin signaling to inhibit expression of gluconeogenic enzymes. Our data indicates that ChREBP-stimulated glucose production supersedes the ability of insulin to suppress it as the effect of ChREBP on gluconeogenic enzyme expression is independent of insulin signaling. This suggests that ChREBP may contribute to the maladaptive phenomenon of “hepatic insulin resistance” that frequently develops early in the pathogenesis of T2D.

**Figure 3:**
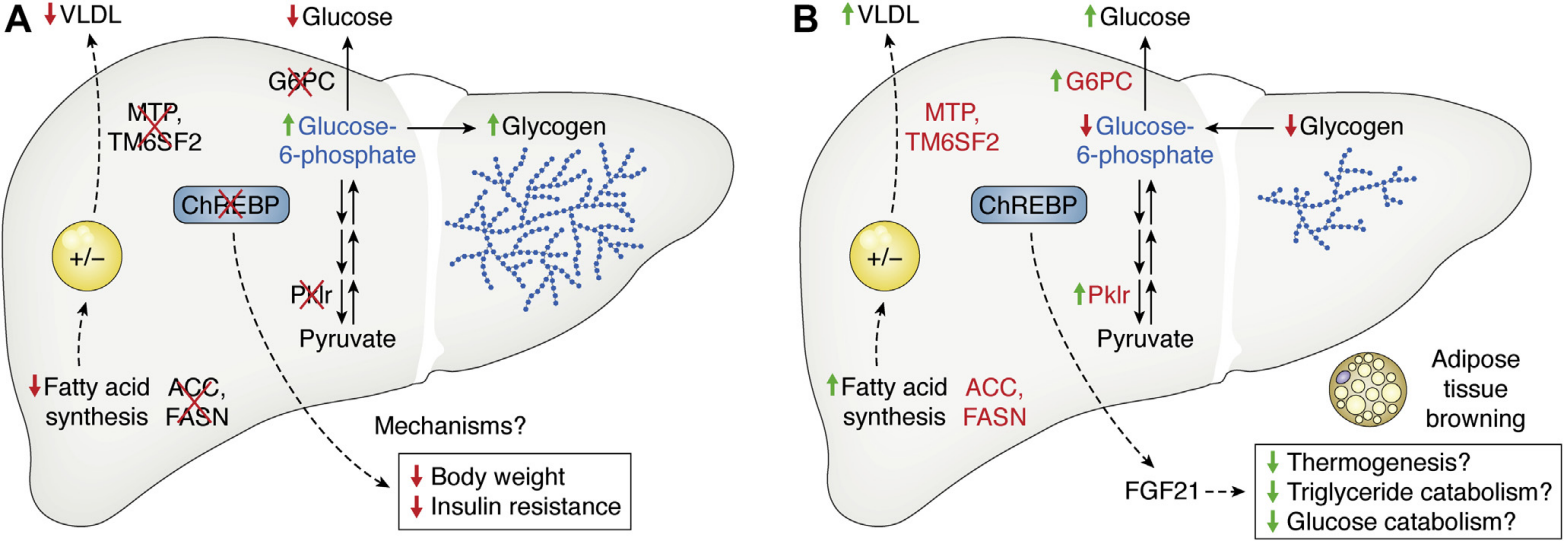
**Liver ChREBP loss- and gain-of-function models increase and decrease hepatocellular G6P and glycogen levels, respectively but produce overlapping phenotypes with respect to systemicfuel metabolism.***A*, liver ChREBP KO reduces the expression of enzymes involved in glycolysis, glucose production, lipogenesis, and VLDL packaging and secretion, which increases hepatocellular G6P and glycogen with variable effects on liver fat content. Through unknown mechanisms, this also reduces body weight and insulin resistance. *B*, liver ChREBP overexpression increases the expression of enzymes involved in glycolysis, glucose production, lipogenesis, and VLDL packaging and secretion, which reduces hepatocellular G6P and glycogen levels with variable effects on liver fat content. ChREBP overexpression also markedly increases circulating FGF21, which has pleiotropic metabolic effects including enhancing browning of white adipose tissue, which may enhance systemic fuel metabolism. Enzymes noted in *red* are ChREBP transcriptional targets.

In addition to a maladaptive role for ChREBP in promoting excessive glucose production, three out of four liver-selective ChREBP loss-of-function studies show that hepatic ChREBP is essential for the development of obesity, hyperinsulinemia, and systemic insulin resistance (Fig. 3*A*) (79, 80, 81, 82). Mechanisms by which hepatic ChREBP activity contributes to these maladaptive features in obesogenic settings are not well understood.

In contrast with the aforementioned studies that show that liver-specific knockout or knockdown of ChREBP protects against obesity and insulin resistance, Jois *et al.* (81) reported that liver-specific ChREBP knockout exacerbates hepatic insulin resistance on both chow and high-fat diets without effects on body weight. In this anomalous study, putative knockout of ChREBP did not reduce the expression of canonical ChREBP transcriptional targets including glycolytic and lipogenic genes. This conflicts with all other ChREBP knockout or knockdown studies including both liver-specific and global ChREBP knockout models as well as knockdown of ChREBP in cell culture. In this study an exon specific for the ChREBP-alpha isoform was selectively deleted potentially leaving the -beta promoter, transcript, and protein intact. All other published loss-of-function studies targeted critical functional domains common to both the ChREBP-alpha and -beta isoforms. This discrepancy suggests that selective loss of hepatic ChREBP-alpha *versus* -beta may produce distinct molecular and metabolic phenotypes. This implies distinct gene regulatory and metabolic functions for the two ChREBP isoforms and will require further investigation.

## Lipid homeostasis in ChREBP loss-of-function models

ChREBP and the insulin-responsive transcription factor SREBP1c synergistically coordinate expression of the enzymes of *de novo* lipogenesis (DNL) (83). ChREBP is essential for DNL enzyme expression and couples the availability of carbohydrate lipogenic precursors to the expression of this program to enhance storage of carbohydrates as fat in the setting of carbohydrate excess. DNL is markedly increased in association with obesity and insulin resistance and is a significant contributor to steatosis—the abnormal accumulation of fat in hepatocytes (84, 85, 86). Yet, while hepatic ChREBP knockout potently impairs hepatic *de novo* lipogenesis, it has variable effects on steatosis. In genetically obese ob/ob mice, RNAi knockdown of ChREBP reduced steatosis (79). In contrast, knockdown or knockout of hepatic ChREBP in dietary models of steatosis had little or no effect on liver triglyceride content (80, 82, 83). ChREBP plays a critical role in VLDL secretion in part through its effects to stimulate expression of key enzymes in this process including Microsomal Triglyceride Transfer Protein (MTTP) and Transmembrane 6 Superfamily, Member 2 (TM6SF2) (74, 80, 87). Preserved steatosis in liver ChREBP KO mice likely occurs due to reduced hepatic VLDL packaging and secretion (80, 83). This is consistent with putative loss-of-function ChREBP variants in humans to reduce circulating triglyceride levels (88).

ChREBP activates lipogenesis and inhibits expression of the transcription factor PPAR-alpha, a master regulator of fatty acid oxidation (89, 90). This is consistent with long-standing observations that lipogenesis and fatty acid oxidation are often regulated in opposition to each other. Nevertheless, recent evidence also indicates that the presence of ChREBP is required for normal PPAR-alpha activity (91). Thus, fatty acid oxidation may be relatively impaired in liver ChREBP KO mice, and this could also contribute to preserved steatosis in these animals despite diminished DNL.

NAFLD appears to be largely a condition of disordered hepatic and systemic lipid metabolism (92). As ChREBP plays a fundamental role in the regulation of hepatic lipid metabolism and fructose consumption may exacerbate NAFLD (93, 94), it is reasonable to speculate that ChREBP may be important in the pathogenesis of NAFLD. ChREBP regulates the expression of at least two enzymes, PNPLA3 and TM6SF2, implicated in the pathogenesis of NAFLD in humans (74, 95). However, neither a specific adaptive nor maladaptive role for ChREBP has been clearly established in the development of steatosis or the progression of steatosis to more advanced forms of NAFLD involving inflammation or fibrosis.

We and others have demonstrated that ChREBP potently transactivates expression of the metabolic hormone FGF21, and FGF21 is under investigation as a therapeutic for treatment of NAFLD (87, 91, 96, 97, 98, 99, 100). FGF21 protects against fructose-induced liver fibrosis, which might support a protective role for ChREBP in the pathogenesis of NAFLD (96). FGF21 also protects against the development of hepatocellular carcinoma in mice on an obesogenic diet (101). However, ChREBP is not required for basal FGF21 expression, and it is unclear whether basal FGF21 is sufficient to provide this protection or whether ChREBP-mediated upregulation of FGF21 is important. Another report suggested that hepatic ChREBP protects against fructose-induced hepatic cholesterol accumulation and the development of severe liver inflammation (47). However, this study used global ChREBP KO mice, which are intolerant to dietary fructose due to intestinal fructose malabsorption, not due to liver toxicity (82, 102, 103). Moreover, in four studies, liver-specific inactivation of ChREBP in rodents challenged with high sucrose or fructose diets produced variable degrees of transaminitis attributable to marked glycogen accumulation, but did not cause hepatic cholesterol accumulation, nor did it cause liver inflammation or fibrosis (76, 80, 82, 83). This phenotype, transaminitis with marked glycogen accumulation, but without development of inflammation or progression to fibrosis or cirrhosis, is reminiscent of aspects of GSD-1a (74, 104, 105). Taken together, these studies show that glycogen accumulation and associated transaminitis occur without inflammation or progressive liver disease in conditions with both increased ChREBP activity in GSD-1a and absent ChREBP activity in ChREBP knockout models. Thus, neither an increase nor absence of ChREBP activity is sufficient to cause liver inflammation or fibrosis under metabolic stress. As a result, a role for ChREBP in the progression of NAFLD remains unclear.

## Hepatic ChREBP gain-of-function models

The majority of ChREBP loss-of-function studies suggest that hepatic ChREBP activity is necessary for sugar and obesity-induced metabolic disease. Paradoxically, two hepatic ChREBP gain-of-function studies suggest that ChREBP can play a protective role (Fig. 3*B*) (87, 97). Adenovirus-mediated overexpression of an N-terminally truncated, constitutively active fragment of ChREBP similar to endogenous ChREBP-beta in the liver lowers circulating glucose, insulin, and triglyceride levels while increasing hepatic *de novo* lipogenesis and steatosis. The beneficial effects of ChREBP overexpression on hepatic insulin sensitivity may be mediated by insulin-sensitizing effects of an increased ratio of monounsaturated fat to saturated fat (87). Nevertheless, the observations that both knockout and overexpression of ChREBP can have positive effects on systemic metabolism has generated significant controversy. Given the translational significance to human health, defining specific mechanisms by which hepatic ChREBP activity may be beneficial or harmful will be essential to resolve this controversy and understand the role for ChREBP and its targets in metabolic health.

Sugar and ChREBP mediated increases in circulating FGF21 signal to the brain to mediate a negative feedback loop to reduce sugar consumption (106, 107, 108). Genome-wide association studies in human populations support this physiology (109, 110). Whereas physiological fluctuations in FGF21 appear to predominately signal through the brain to mediate adaptive changes in food preference, supraphysiological increases in FGF21 or FGF21 agonists, particularly in rodents, cause profound changes in systemic metabolism including weight loss and enhanced systemic glucose and lipid metabolism (111, 112). These improvements in systemic fuel metabolism are associated with browning of white adipose tissue, which may increase energy expenditure and protect against weight gain although other mechanisms are also likely involved (113, 114). Adenovirus ChREBP overexpression markedly increased circulating FGF21 levels in both ChREBP gain-of-function experiments—one of which documented a sevenfold increase in circulating FGF21 within 1 week of viral transduction that was accompanied by browning of white adipose tissue (87, 97). This leads us to speculate that the beneficial effects of overexpressing hepatic ChREBP may be, in part, mediated by sustained supraphysiological increases in FGF21 rather than direct effects of ChREBP to enhance liver insulin sensitivity. The FGF21-mediated benefits on systemic metabolism may mitigate other potentially deleterious effects of increased hepatic ChREBP activity. Again, further investigation will be required to resolve these discrepant observations.

These overexpression experiments indicate that activation of ChREBP in the liver has complex effects on circulating triglycerides. On the one hand, ChREBP contributes to VLDL packaging and secretion, which will increase circulating triglycerides. On the other hand, ChREBP may stimulate production of hepatokines such as FGF21, which may induce clearance of circulating triglycerides. ChREBP overexpression suppressed circulating levels of Angiopoietin-like protein 3 (Angptl3), another hepatokine that inhibits lipoprotein lipase (97, 115). ChREBP-mediated suppression of Angptl3 would also serve to enhance peripheral triglyceride clearance and decrease circulating triglyceride levels. Thus, activation of hepatic ChREBP simultaneously enhances hepatic VLDL production and peripheral clearance. This is potentially consistent with a physiological function to enhance peripheral storage of lipid when carbohydrates are plentiful. ChREBP is likely to regulate additional, as yet undiscovered, hepatokines and signaling molecules that determine ChREBP’s complex effects on systemic metabolism.

While most investigation has focused on the role of ChREBP in regulating systemic lipid and carbohydrate metabolism, recent work also indicates that ChREBP integrates organismal carbohydrate status with amino acid metabolism (116). Adenoviral overexpression of ChREBP-beta in rat liver increases expression of branched-chain ketoacid dehydrogenase kinase (BCKDK) and suppresses expression of protein phosphatase, Mg2+/Mn2+-dependent 1K (PPM1K). BCKDK phosphorylates and inhibits the activity of branched-chain ketoacid dehydrogenase (BCKDH), the rate-limiting step in branched-chain ketoacid catabolism. PPM1K dephosphorylates BCKDH to activate it. ChREBP-mediated inhibition of BCKDH activity may explain the strong association between increased circulating branched-chain amino acids and insulin resistance that is observed in obesity (117). The importance of this mechanism in the pathogenesis of metabolic disease remains to be established.

## Adaptive and maladaptive functions of ChREBP in the pancreatic islet

ChREBP is expressed in pancreatic beta cells at levels that are comparable to those of ChREBP in the liver (118, 119). Since ChREBP responds to glucose metabolites and is a major transcriptional regulator of lipogenesis in the liver, one early hypothesis was that ChREBP in beta cells might participate in glucolipotoxicity-mediated beta cell death. Prolonged hyperglycemia associated with diabetes might stimulate excessive islet DNL and contribute to toxic conditions in beta cells (120, 121, 122, 123). Alternatively, other ChREBP targets might contribute to this toxicity. One such well-defined target is Txnip (thioredoxin interacting protein), which binds to and interferes with thioredoxin, a major antioxidant enzyme in beta cells (124). Depleting beta cells of Txnip protects beta cells from glucotoxic stress. Conversely, overexpression of Txnip in beta cells results in apoptosis (121, 125). Thus, ChREBP, *via* regulation of Txnip, could be a major mediator of glucotoxicity, and this might suggest that ChREBP activation is maladaptive in beta cells. Indeed, recent efforts have targeted beta cell Txnip inhibition to alleviate glucotoxicity and treat diabetes (126, 127).

While excessive beta cell ChREBP activation may be maladaptive, this leaves open the question as to what adaptive, physiological functions ChREBP might mediate in this cell type. Both major forms of diabetes result from insufficient beta cell mass, and therefore major research efforts are underway to identify pathways, interventions, small molecules, and other therapies to expand functional beta cell mass (128). Since glucose itself is a major beta cell mitogen, Metukuri *et al.* (118) tested the hypothesis that ChREBP is required for glucose-stimulated beta cell proliferation. Loss-of-function experiments demonstrated that both rodent and human beta cells require ChREBP for glucose-stimulated beta cell proliferation (56, 118). Moreover, gain-of-function experiments in rodent and human beta cells demonstrated that adenoviral overexpression of ChREBP-alpha augments glucose-stimulated beta cell proliferation without increased apoptosis (118, 129). Wollheim and colleagues observed similar findings in stably transduced Ins-1 cells lines wherein overexpression of SREBP1c, another lipogenic transcription factor, mediates glucose toxicity, but overexpression of full-length ChREBP-alpha had no deleterious effect (130). Thus, ChREBP may promote proliferative expansion of beta cells in an adaptive response to increased demand for insulin.

These initial observations were published before the discovery of distinct ChREBP isoforms. First described in the adipose and liver tissue, ChREBP-alpha and ChREBP-beta are also expressed in beta cells (56). Indeed, as is the case for liver, a robust feed-forward induction of ChREBP exists in beta cells, with an astounding 1000-fold induction of ChREBP-beta mRNA with prolonged exposure to glucose. In beta cells isolated from rat islets and cultured in high-glucose conditions for 4 days, ChREBP-beta expression increases from nearly undetectable levels to levels comparable with that of ChREBP-alpha (56). Furthermore, loss-of-function experiments demonstrate that the induction of ChREBP-beta is necessary for full glucose-mediated induction of ChREBP-dependent gene targets (56). Importantly, the induction of ChREBP-beta is also necessary for glucose-stimulated beta cell proliferation in rodent cell lines and in isolated mouse islet cells (56). These observations show that the induction of ChREBP-beta, which is far more transcriptionally potent than ChREBP-alpha, is the molecular engine that drives glucose-mediated transcription and proliferation in beta cells. Thus, in at least some conditions, ChREBP isoforms have distinct roles in beta cell adaptation.

Gain-of-function experiments testing the effects of the two isoforms have been particularly revealing and may reconcile conflicting observations of ChREBP as a mediator of either adaptive expansion or maladaptive glucose toxicity. Adenoviral overexpression of ChREBP-alpha or doxycycline-mediated induction of ChREBP-alpha does not lead to apoptosis or an immediate decrease in glucose-stimulated insulin secretion (118, 130). Furthermore, despite the necessity for ChREBP-alpha for the feed-forward induction of ChREBP-beta, overexpression of ChREBP-alpha only modestly increases glucose-stimulated expression of ChREBP-beta and does not alter the basal or glucose-induced induction of other ChREBP target genes, including Txnip (129). Kumar *et al.* (129) discovered that overexpression of ChREBP-alpha activates the Nrf2 antioxidant pathway, which promotes proliferation by increasing the expression of antioxidant genes and genes producing NADPH. The latter molecule is necessary for continued antioxidant activity and for the reducing equivalents needed for nucleotide and phospholipid synthesis (131). In stark contrast, ChREBP-beta overexpression, or overexpression of a constitutively active version of ChREBP selectively lacking the LID domain and thus functionally similar to ChREBP-beta, leads to beta cell apoptosis (123, 129). Taken together, these results suggest a unifying hypothesis that the feed-forward induction of ChREBP-beta is necessary for adaptive proliferation of beta cells to meet increased demand for insulin, but excessive ChREBP-beta activity may occur with chronic hyperglycemia in diabetes, which contributes to beta cell apoptosis and glucotoxicity.

It is important to note that Shalev and colleagues demonstrated that ChREBP-beta overexpression decreases ChREBP-alpha abundance and postulated that the role of ChREBP-beta induction in diabetic or glucose-treated beta cells is to prevent ChREBP-alpha from mediating glucose toxicity in a feedback rather than feed-forward regulatory loop (132). However, these experiments did not assess apoptosis and were conducted in relatively short time frames and may have missed the maladaptive effects of ChREBP-beta overexpression. Further experiments with inducible beta cell-specific *in vivo* models are needed to resolve these differences and to test the effects of each isoform on the activity and stability of the other with respect to proliferation, apoptosis, and function *in vivo*.

## Future directions

In aggregate, genetic gain- and loss-of-function models conclusively demonstrate that ChREBP plays both critical adaptive and maladaptive roles in the response to dietary and circulating sugars. The pleiotropic associations between common genetic variants in the ChREBP locus and diverse metabolic traits in human populations further confirm an important role for ChREBP in human metabolic physiology and health. Together, these studies indicate that further investigation into the function of ChREBP will provide a promising avenue to understand the harmful effects of excessive sugar consumption on cardiometabolic risk.

What are the functions of the distinct ChREBP isoforms? Will isoform-specific studies help resolve the apparent discrepancies in the literature to date? To answer these questions, detailed mechanistic investigations with isoform-specific manipulation in each of the key metabolic tissues will be required. Genetic mouse models have only recently become available to begin to approach these isoform-specific questions.

Which ChREBP transcriptional targets contribute to ChREBP’s beneficial *versus* harmful effects? In any of the key metabolic tissues where ChREBP is expressed, it regulates the expression of one to two thousand gene targets (133, 134). The specific metabolic function and physiological role of only a handful of these targets have been investigated to date. Knowledge of the tissue-specific targets that contribute to ChREBP’s beneficial *versus* harmful metabolic effects is necessary to harness the potential of this system for novel diagnostic or therapeutic strategies. Efforts to prioritize ChREBP transcriptional targets that also harbor variants, which associate with metabolic traits in human populations, seem a promising avenue to advance this field.

The overarching goal guiding research in this field over the next decade will be to further understand the mechanisms mediating ChREBP’s beneficial *versus* harmful effects. A detailed knowledge of how ChREBP impacts diverse metabolic traits and cardiometabolic outcomes will need to integrate molecular mechanistic studies performed *in vitro* and in animal models with physiological or population genetics approaches in humans. To harness this promise will require substantial investment of time and resources to generate novel reagents to test relevant hypotheses.

## Conflict of interest

The authors declare that they have no conflicts of interest with the contents of this article.
